# Association of *p53* codon 72 polymorphism with advanced lung cancer: the Arg allele is preferentially retained in tumours arising in Arg/Pro germline heterozygotes

**DOI:** 10.1038/sj.bjc.6600595

**Published:** 2002-10-21

**Authors:** E D Papadakis, N Soulitzis, D A Spandidos

**Affiliations:** Laboratory of Virology, Medical School, University of Crete, PO Box 1393, Heraklion, Crete, Greece

**Keywords:** *p53*, codon 72 polymorphism, lung cancer, human papillomavirus, loss of heterozygosity

## Abstract

The association of *p53* codon 72 polymorphism with cancer has been investigated by several scientific groups with controversial results. In the present study, we examined the genotypic frequency of this polymorphism in 54 patients with advanced lung cancer and 99 normal controls from the geographical region of Greece. Sputum and bronchial washing samples from each patient were assayed for the presence of human papillomavirus. Codon 72 heterozygous (Arg/Pro) patients were also analysed for loss of heterozygosity at the *TP53* locus, in order to determine the lost *p53* allele (Arg or Pro). *p53* Arg/Arg genotype was significantly increased in lung cancer patients compared to normal controls (50% *vs* 24.2%, *P*<0.002). Human papillomavirus was detected only in two patients (3.7%). Loss of heterozygosity at the *TP53* locus was found in 14 out of 27 Arg/Pro patients (51.85%). The Pro allele was lost in 11 cases (78.6%), while the Arg allele was lost in three (21.4%). Our results suggest that *p53* codon 72 Arg homozygosity is associated with advanced lung cancer, and that the Arg allele is preferentially retained in patients heterozygous for this polymorphism. On the other hand, human papillomavirus infection does not seem to play an important role in lung carcinogenesis.

*British Journal of Cancer* (2002) **87**, 1013–1018. doi:10.1038/sj.bjc.6600595 www.bjcancer.com

© 2002 Cancer Research UK

## 

Lung cancer is the most common cause of cancer-related deaths in Greece, accounting for 574 deaths per million people per year (Jemal *et al*, 2002). Radical treatment depends primarily on early detection, since patients with advanced and metastatic disease have 5-year survival rates less than 5% (Hirsch *et al*, 2001). Identification of early molecular events in lung carcinogenesis, such as *ras* gene mutations or *p53* inactivation, could have a major impact on early detection of this disease (Viallet and Minna, 1990).

Human papillomavirus (HPV) has been implicated in the development of several human cancers, including cervical, oesophageal, skin, and laryngeal cancer (zur Hausen, 1996). Among the proteins encoded in the HPV genome, early protein E6 binds *p53* and promotes its degradation through the ubiquitin-dependent proteolytic pathway (Scheffner *et al*, 1990, 1993). HPV DNA is detected in less than 10% of lung cancer patients (Kinoshita *et al*, 1995; Bohlmeyer *et al*, 1998; Clavel *et al*, 2000), suggesting that HPV does not play an important role in the development of lung cancer.

The human *p53* tumour suppressor gene is located on chromosome 17p13 and encodes a 53-kDa nuclear phosphoprotein, which plays a central role in many cellular processes, such as DNA repair and apoptosis (Levine, 1997). Allelic loss at the *TP53* locus is observed in more than 50% of human cancers, disclosing aberrations, like missense point mutations, in the retained allele (Hollstein *et al*, 1991). The *p53* gene is mutated in more than 50% of lung cancer cases (Takahashi *et al*, 1991).

*p53* exists in two principal polymorphic forms that have either arginine (*p53Arg*) or proline (*p53Pro*) at codon 72 (Matlashewski *et al*, 1987). It has been proposed that *p53Arg* protein is more susceptible to inactivation through the E6-ubiquitin pathway than the *p53Pro* isoform, and that *p53* Arg/Arg homozygosity is associated with increased risk of developing HPV-associated cervical cancer (Storey *et al*, 1998). Similar studies on cervical and other human cancers have produced contradictory results. Several groups confirmed the original finding (Makni *et al*, 2000; Zehbe *et al*, 2001), some have shown that the association between *p53* Arg/Arg genotype and cancer is unrelated to HPV (Andersson *et al*, 2001), while others failed to find an association between *p53Arg* and cancer (Hamel *et al*, 2000; Tenti *et al*, 2000). In lung cancer, several studies found no correlation with *p53* codon 72 polymorphism (Birgander *et al*, 1995; Pierce *et al*, 2000), while others have found an association with the Pro allele (Kawajiri *et al*, 1993; Wang *et al*, 1999; Fan *et al*, 2000). However, in tumours with loss of heterozygosity (LOH) at the *TP53* locus, the Pro allele is preferentially deleted in germline Arg/Pro heterozygotes (Brooks *et al*, 2000; Kawaguchi *et al*, 2000; Furihata *et al*, 2002).

In this study we examined the genotypic frequency of *p53* codon 72 polymorphism and the presence of HPV infection in 54 patients with advanced lung cancer, compared to a healthy control population, in order to identify their association with lung carcinogenesis. Furthermore, using microsatellite analysis, we investigated the frequency of LOH at the *TP53* locus, in patients heterozygous for the codon 72 polymorphism, in order to determine possible preferential retention of either Arg or Pro allele.

## MATERIALS AND METHODS

### Patients and controls

Fifty-four Greek Caucasian patients with advanced lung cancer (Stages IIIb and IV) were recruited during the diagnostic workup for lung cancer in the Department of Pulmonary Medicine, University Clinic of Sotiria Hospital (Athens, Greece). Cancer staging was performed with CT scan and fiberoptic bronchoscopy. Mean age at diagnosis was 66.4±8.3 years, with men accounting for 85.2% of cases. Patients were heavy smokers with an average of 64.6±31.4 pack-years (Table 1

**Table 1:**
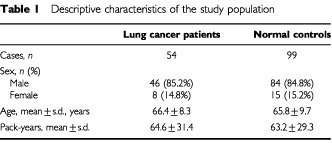
Descriptive characteristics of the study population

). Histologically, cancer cases comprised 23 adenocarcinomas, 24 squamous cell carcinomas, six small cell lung carcinomas and one large cell carcinoma. Cytological specimens of sputum and bronchial washing were obtained from each patient and stored at −80°C until DNA extraction. Matched peripheral blood samples were collected in tubes containing EDTA and stored at 4°C, to serve as source of genomic DNA.

Ninety-nine Greek Caucasian healthy individuals served as the control group. They exhibited similar age, sex and smoking exposure distribution with patients (Table 1) and derived from the same geographical area. Peripheral blood samples were obtained from each individual and stored at 4°C for further use.

The Ethics Committees of the University of Crete and the University Clinic of Sotiria Hospital approved this study and all participants gave written informed consent.

### DNA extraction

Sputum, bronchial washing and peripheral blood samples from patients as well as peripheral blood samples from controls were lysed with 400 mM Tris-HCl pH 8.0, 150 mM NaCl, 60 mM EDTA and 1% SDS, adding 50 μg Proteinase K and incubated for 5 h at 65°C.

DNA from all specimens was extracted with phenol/chloroform and chloroform, precipitated with absolute ethanol, washed with 70% ethanol and resuspended in 100 μl double distilled water.

The presence of amplifiable DNA was tested in a PCR reaction using a set of primers for the β2-microglobulin (β2-m) gene (Table 2

**Table 2:**
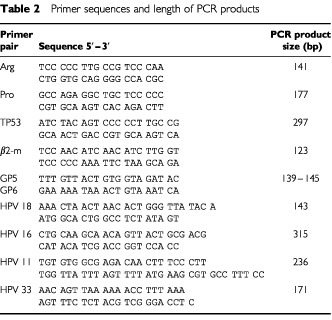
Primer sequences and length of PCR products

).

### *p53* codon 72 polymorphism analysis

For the determination of the polymorphism at codon 72 of the *p53* gene, an allele-specific PCR assay was used (Storey *et al*, 1998), that selectively detects either the Arg or Pro *p53* allele (Table 2). A total of 100 ng of peripheral blood DNA from patients and controls was amplified in a PCR reaction containing 1× buffer (20 mM Tris-HCl pH 8.4 and 50 mM KCl), 1.6 mM MgCl_2_, 200 μM of each dNTP, 200 nM of each primer and 0.024 U μl^−1^ Taq polymerase (Gibco BRL, Invitrogen Corporation) in a final volume of 25 μl. Detection of the two polymorphic variants was carried out in two different tubes. Amplification was performed as follows: initial denaturation at 94°C for 3 min, amplification for 35 cycles at 94°C for 30 s, 60°C for the Arg allele and 54°C for the Pro allele for 30 s and 72°C for 30 s, followed by a final elongation step at 72°C for 5 min. PCR products were 141 bp for the Arg allele and 177 bp for the Pro allele. Heterozygous specimens had both PCR products, whereas homozygous samples exhibited only one of the two products. In each PCR reaction two blank samples were employed as negative controls, to ensure that no contaminants were introduced. PCR products were analysed by electrophoresis in a 2% agarose gel, stained with ethidium bromide and photographed on a UV light transilluminator. Procedures were repeated twice and results were 100% reproducible.

This analysis was also conducted in bronchial washing specimens from heterozygous (Arg/Pro) patients with LOH at the *TP53* locus. Faint bands in the position of the lost allele were interpreted as DNA contamination from normal lung cells.

### LOH analysis at the *TP53* locus

For the determination of LOH at the *TP53* locus, the TP53 microsatellite marker set was used (Table 2). A total of 100 ng of peripheral blood and bronchial washing DNA from each heterozygous (Arg/Pro) patient was amplified in a PCR reaction containing 1× buffer, 2.66 mM MgCl_2_, 500 μM of each dNTP, 100 nM of each primer and 0.04 U μl^−1^ Taq polymerase in a final volume of 25 μl. Amplification was carried out as follows: initial heating at 94°C for 3 min, amplification for 30 cycles at 94°C for 30 s, 55°C for 30 s and 72°C for 30 s and final elongation at 72°C for 10 min. PCR products were analysed by electrophoresis in a 10% polyacrylamide gel and silver stained. LOH was scored when a significant reduction (>50%) in the intensity of one allele was observed. Most LOH cases exhibited faint bands in the position of deleted alleles, which was interpreted as contamination with normal DNA. Analysis in LOH positive cases was repeated at least twice and results were highly reproducible.

### HPV detection and typing

For the detection of HPV genome, the general primers GP5 and GP6 were used (Table 2). A total of 100 ng of sputum and bronchial washing DNA from each patient was amplified in a PCR reaction containing 1× buffer, 1.6 mM MgCl_2_, 200 μM of each dNTP, 200 nM of each primer and 0.024 U μl^−1^ Taq polymerase in a final volume of 25 μl. The mixture was heated at 94°C for 3 min and samples were amplified for 40 cycles at 94°C for 30 s, 52°C for 30 s and 72°C for 30 s, followed by elongation at 72°C for 5 min. PCR products were expected to be 139–145 bp, depending on HPV type (Snijders *et al*, 1990).

HPV typing of all HPV-positive samples was carried out using multiplex PCR. Specific pairs of primers were used to simultaneously amplify regions of HPV types 11, 16, 18 and 33 in the same reaction tube, giving different lengths of amplified DNA. Each HPV-positive sputum or bronchial washing sample was amplified in a PCR reaction containing 1× buffer, 2.66 mM MgCl_2_, 0.05% W-1, 533 μM of each dNTP, 166 nM of each primer and 0.04 U μl^−1^ Taq polymerase in a final volume of 15 μl. Amplification parameters were: initial heating at 94°C for 2 min and 30 s, 10 cycles of amplification at 94°C for 30 s, 52°C for 40 s and 72°C for 45 s, 30 cycles of amplification at 92°C for 30 s, 48°C for 40 s and 72°C for 45 s, and final extension at 72°C for 10 min. To establish type specificity of primer-directed amplification, each set of primers was tested with template plasmid DNA of the four HPV types (11, 16, 18 and 33). PCR products were analysed by electrophoresis in a 2% agarose gel, stained with ethidium bromide and photographed on a UV light transilluminator. Primer sequences and length of PCR products are shown in Table 2.

### Statistical analysis

Statistical analysis was performed using the χ^2^, Fisher's Exact and Mann–Whitney U tests, with the package SPSS 10.0 for Windows. Statistical significance was regarded at *P*-value <0.05.

## RESULTS

### *p53* Arg is associated with advanced lung cancer

Peripheral blood DNA from 54 patients with advanced lung cancer was analysed, in order to determine the distribution of *p53* codon 72 polymorphism. In order to detect any discrepancy in the frequency of this polymorphism among cancer patients and the general population, peripheral blood specimens from 99 healthy individuals were subjected to the same PCR-based genotyping assay.

Distribution of the three genotypes, Arg/Arg, Arg/Pro and Pro/Pro, was 50%, 50% and 0% in lung cancer patients, and 24.2%, 64.7% and 11.1% in healthy controls respectively (Table 3

**Table 3:**
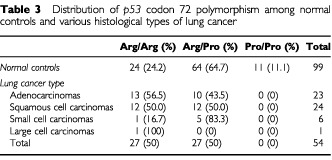
Distribution of *p53* codon 72 polymorphism among normal controls and various histological types of lung cancer

). Based on the above results, the relative frequency of each allele was 0.75 for Arg and 0.25 for Pro in cancer patients, and 0.565 for Arg and 0.435 for Pro in normal controls, indicating a statistically significant difference (χ^2^=7.99, *P*<0.005, odds ratio (OR)=2.36, 95% confidence intervals (CI)=1.24–4.50). Further analysis revealed that the frequency of Arg/Arg genotype was considerably higher in patients than in controls (χ^2^=10.43, *P*<0.002, OR=3.13, 95% CI=1.46–6.73). This association was also observed separately in adenocarcinomas (χ^2^=9.20, *P*<0.003, OR=4.06, 95% CI=1.44–11.62) and squamous cell carcinomas (χ^2^=6.19, *P*<0.02, OR=3.13, 95% CI=1.13–8.69), but not in small cell lung carcinomas (Fisher's Exact test, *P*=0.56) or in large cell carcinomas (Fisher's Exact test, *P*=0.25).

Interestingly, both study groups (patients and controls) showed a deviation from the Hardy–Weinberg equilibrium due to excess of heterozygotes (Controls: χ^2^_HW_=9.86, f=1, *P*<0.002; Patients: χ^2^_HW_=6.00, f=1, *P*<0.02).

Patients were then stratified according to age, sex, histological classification and smoking exposure (in pack-years). A significant association of Arg/Arg adenocarcinomas with lower number of pack-years was observed, compared to Arg/Pro adenocarcinomas (30±8.5 *vs* 65±8.5) (Mann–Whitney U test, Z=−4.073, *P*<0.001). No other statistically significant difference was found between the codon 72 polymorphism and the other variables examined.

### *p53* Pro is preferentially deleted in Arg/Pro germline heterozygotes

**Figure 1:**
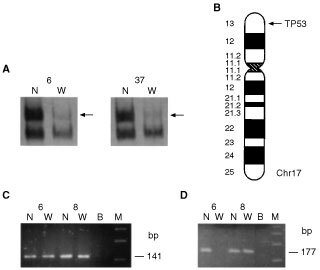
(**A**) Representative examples of specimens exhibiting LOH at the *TP53* locus. N; DNA from peripheral blood lymphocytes. W; DNA extracted from bronchial washing. Arrows indicate the missing allele. The upper row indicates patient code number. (**B**) Location of the TP53 microsatellite marker at chromosome 17p13. (**C**) PCR amplification of *p53* codon 72 Arg allele (141 bp). N; DNA from peripheral blood lymphocytes. W; DNA extracted from bronchial washing. B; blank sample. M; 123 bp molecular weight marker. The upper row indicates patient code number. (**D**) PCR amplification of *p53* codon 72 Pro allele (177 bp). N; DNA from peripheral blood lymphocytes. W; DNA extracted from bronchial washing. B; blank sample. M; 123 bp molecular weight marker. The upper row indicates patient code number.

Lung cancer patients heterozygous for the codon 72 polymorphism (27 in total) were examined for LOH at the *TP53* locus. Matched DNA samples from peripheral blood and bronchial washing were analysed, using the TP53 microsatellite marker (Figure 1B

). A total of 14 of 27 Arg/Pro patients (51.85%) exhibited LOH (Figure 1A). In order to identify the deleted *p53* allele, the 14 bronchial washing samples with LOH were analysed for the codon 72 polymorphism (Figure 1C, D). The Arg allele was preferentially retained, as it was lost only in three (21.4%) cases, whereas the Pro allele was lost in 11 (78.6%) cases (Table 4

**Table 4:**
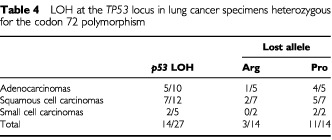
LOH at the *TP53* locus in lung cancer specimens heterozygous for the codon 72 polymorphism

).

### HPV infection is a rare event in lung carcinogenesis

**Figure 2:**
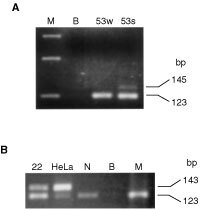
(**A**) Detection of HPV by PCR amplification using the general primers G5 and G6 (145 bp). 53w; bronchial washing. 53s; sputum. B; blank sample. M; 123 bp molecular weight marker. The band at 123 bp corresponds to the PCR product of β2-m gene, which was used to establish the presence of amplifiable DNA. (**B**) Detection of HPV-18 by PCR amplification (143 bp). 22; patient sample positive for HPV-18. HeLa; positive control from HeLa cells. N; negative control. B; blank sample. M; 123 bp molecular weight marker. The band at 123 bp corresponds to the PCR product of β2-m gene, which was used to establish the presence of amplifiable DNA.

In order to determine the frequency of HPV in advanced lung cancer, and to find a possible association with *p53* codon 72 polymorphism, matched biological specimens, from the upper (sputum) and lower (bronchial washing) respiratory system of each patient, were analysed for the presence of HPV, using the general primers GP5 and GP6. HPV DNA was detected in two cases (3.7%), a squamous cell carcinoma and a large cell carcinoma. However, in the patient with squamous cell carcinoma, HPV was detected only in sputum and not in bronchial washing (Figure 2A

). Multiplex PCR revealed that both patients harboured the high-risk HPV-18 type (Figure 2B). Although the Arg/Arg genotype of *p53* codon 72 polymorphism was observed in both HPV-positive cases, this finding was not statistically significant (Fisher's Exact test, *P*=0.25).

## DISCUSSION

This population-based case-control study was conducted to examine the prevalence of *p53* codon 72 polymorphism in a Greek Caucasian population of advanced lung cancer patients and normal controls. The Arg/Arg homozygous genotype was statistically higher in patients than in controls (50% *vs* 24.2%, *P*<0.002). This finding is in accordance with the original publication on HPV-associated cervical cancer (Storey *et al*, 1998), with previous studies by our group in cervical (Dokianakis and Spandidos, 2000), breast (Papadakis *et al*, 2000), skin (Dokianakis *et al*, 2000), laryngeal (Sourvinos *et al*, 2001) and bladder cancer (Soulitzis *et al*, 2002), as well as with another Greek study in cervical intra-epithelial neoplasia and cervical cancer (Agorastos *et al*, 2000).

Both study groups showed deviation from the Hardy-Weinberg equilibrium (Controls: χ^2^_HW_=9.86, *P*<0.002; Patients: χ^2^_HW_=6.00, *P*<0.02). Several studies exhibit this deviation in the control (Jin *et al*, 1995; Makni *et al*, 2000) or patients group (Kawajiri *et al*, 1993; Tandle *et al*, 2001). This cannot be attributed to methodological problems. The technique used to type this polymorphism (i.e. allele specific PCR), has a tendency to under-estimate heterozygotes (if an allele specific PCR product is not found, the specimen is characterised as homozygous for the other allele). On the contrary, the method that uses analysis with restriction enzymes, has a tendency to over-estimate heterozygotes (due to incomplete digestion of PCR products from the restriction enzyme). Therefore, regarding the cancer population, the Hardy-Weinberg equilibrium deviation could be attributed to selection bias due to the disease itself. As for the control group, it could be attributed to non-random selection, since enlisted subjects had specific characteristics (age, sex and smoking exposure) matching those of the cancer group. Nevertheless, from a statistical point of view, this Hardy-Weinberg equilibrium deviation does not affect our findings. Calculating the theoretical genotype frequencies for both controls and patients, the increase of Arg/Arg genotype in patients is statistically significant (χ^2^=9.08, *P*<0.003, OR=2.82, CI=1.35–5.94). These values are only slightly lower than the ones calculated with the observed genotypic frequencies (χ^2^=10.43, *P*<0.002, OR=3.13, CI=1.46–6.73).

The frequency of Pro allele in the control group is 43.5%. It has been proposed that geographical location and ethnicity play an important role in *p53* allele frequencies (Beckman *et al*, 1994). Distribution of Pro allele in Caucasian populations varies from 24% (Finland) (Beckman *et al*, 1994) to 53% (India) (Tandle *et al*, 2001). Therefore, the Pro allele frequency of this study is not high, taking into account the geographical location of Greece and the genetic characteristics of our population. Furthermore, it is similar to that found by another Greek study (48%) (Agorastos *et al*, 2000) and by a Turkish (a proximate population) study (39%) (Toruner *et al*, 2001).

Several scientific groups have drawn opposite conclusions, associating *p53Pro* with lung carcinogenesis. This discrepancy could be attributed to various reasons. In some studies cancer population comprised patients eligible for surgical treatment (Stages I and II) excluding patients with more advanced tumours (Fan *et al*, 2000), whereas all of our cases were inoperable lung cancers (Stages IIIb and IV). Additionally, in studies involving advanced tumours, statistical analysis did not reveal any association of *p53Pro* with lung carcinogenesis (Wang *et al*, 1999). Moreover, variations in protocols among laboratories, or poor selection of the control group (Makni *et al*, 2000), could also explain the above mentioned discrepancies. However, it seems that the main reason is ethnic background. Populations across the earth exhibit significant differences in the frequency of *p53* codon 72 polymorphism, which can be attributed to natural selection through ecological adaptation to ultra-violet radiation (Beckman *et al*, 1994). Most studies that associate *p53Pro* with lung cancer have been conducted in Southeast Asia (China, Taiwan or Japan), where another *p53* polymorphism (a 16 bp duplication in intron 3) is almost absent (frequency <1.5%). This 16 bp duplication, combined with the Pro/Pro codon 72 genotype, offers protection against lung cancer. Therefore, the low frequency of the protective 16 bp duplication in these populations would make it easier to demonstrate an association between *p53Pro* and cancer (Birgander *et al*, 1995).

Statistical analysis revealed that patients with Arg/Arg adenocarcinoma have lower pack-years of smoking history than those with Arg/Pro adenocarcinoma (30 *vs* 65, *P*<0.001). This finding suggests that, as *p53Arg* confers susceptibility to lung carcinogenesis, lower dose of the carcinogen (tobacco) is required for tumour development. Larger studies, including phase I (*CYPs*) and phase II (*GSTs* and *NATs*) detoxification enzymes, are needed to verify this preliminary result.

Several functional assays demonstrate that the two polymorphic variants of *p53* have different biochemical and biological characteristics (Storey *et al*, 1998; Thomas *et al*, 1999; Marin *et al*, 2000). Codon 72 lies within a proline-rich domain of *p53* required for growth suppression (Walker and Levine, 1996) and apoptosis (Sakamuro *et al*, 1997). It comprises five PxxP SH3 (SRC-homology-3) binding motifs, one of which is lost when proline is replaced with arginine. This substitution could account for the difference that these two *p53* variants exhibit in E6-mediated degradation, transcription activation and induction of apoptosis, and could explain the implication of codon 72 polymorphism in carcinogenesis.

Of the 14 heterozygous (Arg/Pro) specimens with LOH at the *TP53* locus, 11 (78%) retained the Arg allele, while only three (22%) retained the Pro allele. Preferential deletion of *p53Pro* has also been described in HPV-associated oesophageal cancer (Kawaguchi *et al*, 2000), in squamous cell carcinoma of the vulva, regardless of HPV infection (Brooks *et al*, 2000) and in transitional cell carcinoma of the urinary tract (Furihata *et al*, 2002). The Arg isoform of *p53* codon 72 enhances certain *p53* mutants to form stable complexes with *p73*, blocking the apoptotic ability of *p73* (Marin *et al*, 2000). Therefore, *p53* mutants carrying the Arg allele can lead to decreased activation of *p53* target genes through inactivation of *p73* (Tada *et al*, 2001). Thus, there is a strong bias to mutate and retain the Arg allele in tumours arising in Arg/Pro germline heterozygotes.

HPV was detected in two (3.7%) lung cancer patients, a squamous cell carcinoma and a large cell carcinoma. The low frequency of HPV infection is consistent with previous studies performed in lung cancer (Kinoshita *et al*, 1995; Bohlmeyer *et al*, 1998; Clavel *et al*, 2000), suggesting that HPV is not an important factor in lung tumorigenesis. However, it is possible that the effect of HPV infection can only be estimated when other important carcinogenic factors, such as tobacco exposure, are not present (Cheng *et al*, 2001). Typing assay revealed that both cases harboured HPV-18. The high prevalence of this oncogenic HPV type in the Greek population could be attributed to ethnic variations (Dokianakis *et al*, 1999). In the case of squamous cell carcinoma, HPV DNA was detected only in sputum and not in bronchial washing. This could mean either that HPV infection was important solely as an early event in carcinogenesis and persistence of viral DNA was not necessary for cancer progression (Brooks *et al*, 2000), or that sputum was contaminated with cells from the upper respiratory tract or the oral cavity, infected with HPV. Both cases carried the Arg/Arg codon 72 genotype. Nevertheless, due to the low frequency of HPV infection, the association of *p53Arg* with lung carcinogenesis was not related to HPV.

In conclusion, our data associate *p53Arg* with advanced lung cancer and support the observation, that the Arg allele is preferentially retained in Arg/Pro germline heterozygotes. Nonetheless, additional population-based and functional studies are needed in order to elucidate the role of *p53* codon 72 polymorphism in lung cancer development.
